# Supplementary material for: Advanced Glycation End-Products Suppress Mitochondrial Function and Proliferative Capacity of Achilles Tendon-Derived Fibroblasts
Source: Sci Rep. 2019 Aug 30;9:12614. doi: 10.1038/s41598-019-49062-8 (PMC6717202; doi:10.1038/s41598-019-49062-8)
Supplement: Supplementary file 1 — Supplementary Information [file 41598_2019_49062_MOESM1_ESM.pdf]

Supplementary Information

Advanced Glycation End-Products Suppress Mitochondrial Function and Proliferative Capacity of Achilles Tendon-Derived Fibroblasts

Shivam H. Patel[1]
Feng Yue[2]
Shannon K. Saw[1]
Rachel Foguth[3,4]
Jason R. Cannon[3,4]
Jonathan H. Shannahan[3]
Shihuan Kuang[2]
Arman Sabbaghi[5]
Chad C. Carroll[1,6*]

[1]Department of Health and Kinesiology, Purdue University, West Lafayette, IN
[2]Department of Animal Sciences, Purdue University, West Lafayette, IN
[3]School of Health Sciences, Purdue University, West Lafayette, IN
[4]Purdue Institute for Integrative Neuroscience, West Lafayette, IN
[5]Department of Statistics, Purdue University, West Lafayette, IN
[6]Indiana Center for Musculoskeletal Health, Indiana University School of Medicine, Indianapolis, IN

*Address for correspondence:

Chad C. Carroll, PhD
Assistant Professor
Purdue University
Department of Health and Kinesiology
800 W. Stadium Ave
West Lafayette, IN 47907
Phone: (765) 496-6002
carrol71@purdue.edu

Full Unedited Blots for Figure 7

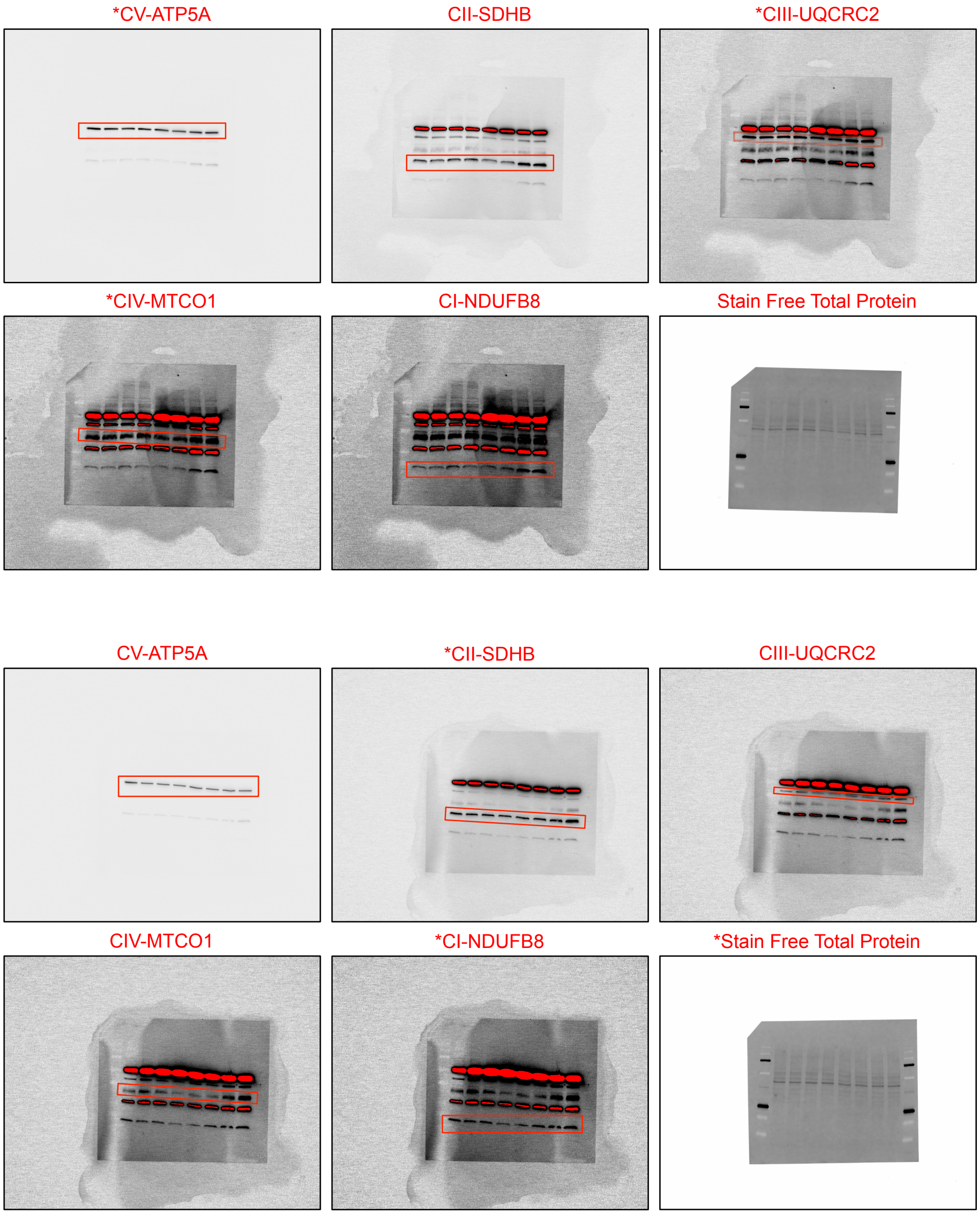

Supplementary Figure 1. Two full and unedited blots for Figure 7. All five targets were probed on the same membrane, but required different exposure times using signal accumulation mode (ChemiDoc, BioRad). Blot images are shown in order of exposure time and (*) indicates the image that was cropped and used for representative purposes in Figure 7f.
